# Self-compassion as predictor of daily physical symptoms and chronic illness across older adulthood

**DOI:** 10.1177/13591053211002326

**Published:** 2021-03-27

**Authors:** Heather Herriot, Carsten Wrosch

**Affiliations:** Concordia University, Canada

**Keywords:** advanced old age, aging, chronic illness, daily physical symptoms, self-compassion

## Abstract

This study examined whether self-compassion could benefit daily physical symptoms and chronic illness in early and advanced old age. The hypotheses were evaluated in a 4-year longitudinal study of 264 older adults. Results showed that self-compassion predicted lower levels of daily physical symptoms across the study period in advanced, but not early, old age (*T-ratio* = −1.93, *p* = 0.05). In addition, self-compassion was associated with fewer increases in chronic illness in advanced, but not early, old age (*T-ratio* = − 2.45, *p* < 0.02). The results of this study suggest that self-compassion may be particularly adaptive towards the end of life.

## Introduction

While the length of the human life span has increased substantially, the health of older adults during these added years has not kept up proportionally. Older adults generally experience a decline in their health that can be due to the rise of both daily physical symptoms and chronic health problems (Gerstorf et al., 2013; Wrosch and Schulz, 2008). The prevalence of physical health problems generally increases as individuals shift from early to advanced old age. In early older adulthood, for example, less than half of older adults in Canada report having at least one chronic illness, but by age 71 and greater, over 80% of older adults report having at least one chronic illness (Statistics Canada, 2009). Daily physical symptoms may be a sign of manifesting disease or a precursor of developing chronic illness and these health problems can lead to a multitude of negative downstream consequences associated with poor psychological well-being, disability, or mortality (Freund and Baltes, 2000; Williamson and Schulz, 1995; Wrosch and Schulz, 2008). It is therefore important to focus on psychological factors that could protect health as older adults advance in age (Baltes and Smith, 2003; Smith et al., 2002).

One area of theory and research addresses psychological factors that may facilitate coping with age-related stressors. To this end, motivational theories of life-span development provide a theoretical context to understand which psychological variables are potentially adaptive as individuals advance in age (e.g. Brandtstädter and Renner, 1990; Freund and Baltes, 2000; Heckhausen et al., 2019). With increasing age, people experience a decline in internal resources, opportunities, and control available to overcome stressors (Heckhausen et al., 2019). As a consequence, older adults may need to increasingly rely less on processes that involve attempts at overcoming stressors and attaining those goals that have become uncontrollable or unattainable (e.g. persistence, Wrosch et al., 2000). Instead, as older adults move from early to advanced old age, they may need to shift towards self-protective strategies that aim to regulate negative emotions and promote disengagement from unattainable goals (e.g. positive reappraisals; Heckhausen et al., 2019; Jobin and Wrosch, 2016; Wrosch et al., 2006).

Self-compassion may represent one of these self-protective factors that we theorize may become increasingly adaptive during older adulthood. Self-compassion can be conceptualized as a dispositional factor that involves treating yourself in the same kind, caring, and compassionate manner that a person would treat a close friend or loved one who experiences stress (Neff, 2003a). To be self-compassionate involves being able to recognize the experience of stress or failure in a mindful manner with-out criticizing, blaming or excessively rumi-nating on the experience (Neff, 2003b). Self-compassionate individuals are also less likely to feel alone in their experiences of stress and failure as they are more likely to contextualize problematic experiences as common to the human condition (Neff, 2003b). These psychological concomitants of self-compassion are likely to support health-relevant processes, such as well-being, among older adults who experience stressful life circumstances. In support of this assumption, a recent meta-analysis indicated that self-compassion is associated with better psychological well-being in older adults (Brown et al., 2019).

Research has also studied whether self-compassion may benefit physical health. For example, a recent meta-analysis of mostly adult samples suggested that higher self-compassion does generally predict better physical health (Phillips and Hine, 2019). In addition, self-compassion has been related to objective biological markers of health. A cross-sectional study from our laboratory has shown that self-compassion may protect older adults who report age-related stressors from enhanced daily cortisol output (Herriot et al., 2018). In addition, a randomized controlled trial of adult patients with diabetes demonstrated that promoting self-compassion buffered the effects of stress on metabolic indicators of diabetes control (Friis et al., 2015).

Self-compassion may promote better health through a variety of pathways. For example, self-compassion is likely to facilitate more positive health behaviors in the context of stressors, such as better eating behavior, sleep habits, physical activity, and medication compliance (Terry and Leary, 2011). The association between self-compassion and health behavior, however, seems to become less pronounced in older adulthood (Phillips and Hine, 2019). Self-compassion may also improve health by reducing the severity of stress experiences. For example, self-compassion has been shown to be associated with more adaptive coping responses (e.g. Allen and Leary 2010), which could facilitate more adaptive biological reactivity to stress (e.g. cortisol and inflammation; Breines et al., 2014a, 2014b; Herriot et al., 2018). Since dysregulated cortisol secretion can influence other health-relevant bodily systems (e.g. immune function, Cohen et al., 2007), it is plausible to assume that self-compassion may also slow down the development of a number of daily physical symptoms and chronic diseases in older adulthood (cf. Wrosch et al., 2004). As a consequence, the pathway linking self-compassion to better health could be related to processes that promote stress-reduction in older adulthood.

One limitation of the extant literature is its reliance on cross-sectional research, and the limited exploration of these associations during older adulthood. As such, there is a paucity of work on the association between self-compassion and changes in physical health among older adults. To date, no research has studied whether self-compassion could predict the longitudinal development of either daily or chronic health problems among older adults. In addition, this literature has focused on older adults collectively, and there is a lack of research exploring possible age differences in the effects of self-compassion among people in early versus advanced old age (e.g. Phillips and Hine, 2019). As discussed previously, motivational life-span theories would assume that individual difference factors that support self-protection and facilitate disengagement from unattainable goals are sensitive to a person’s age-related context and become particularly adaptive in advanced old age, when desired goals become frequently unattainable and individuals confront an increasing number of uncontrollable stressors (Baltes and Smith, 2003; Heckhausen et al., 2019). In early old age, by contrast, when many individuals still have sufficient opportunities to overcome stress experiences, processes other than self-compassion (e.g. persistence, Heckhausen et al., 2019) may be more important for health-related functioning, and the health effects of self-compassion could be comparatively reduced. Consistent with this assumption, research has shown that the beneficial effects of self-protective factors on preventing depressive symptom and physical disease increased from early to advanced old age (e.g. Jobin and Wrosch, 2016). Since self-compassion may exert similar buffering effects, it may thus also become paramount for protecting physical health in advanced old age.

To study this possibility, the present study used longitudinal data from a community-dwelling sample of older adults to test if self-compassion can predict levels and trajectories of common physical health outcomes over time, such as daily physical symptoms and chronic illness (Gerstorf et al., 2013). Given that self-compassion may be health-protective particularly when individuals confront an increasing number of uncontrollable stressors and unattainable goals, we further hypothesized that beneficial effects of self-compassion on physical health outcomes would be enhanced in advanced, as compared to early, old age.

## Method

### Participants

Data for this study involved community-dwelling older adults from the Montreal Aging and Health Study (MAHS). Participants were recruited via newspaper advertisements in the Montreal area. At T1 215 participants were originally assessed, and at T6 the sample was refreshed to include a total of 268 participants (95 original and 173 new participants). Only those who participated at T6 were considered for participation given that the primary measure of interest (i.e. self-compassion) was not assessed in the study until T6. As a result, we only used data from T6 onward, which was considered baseline for the purpose of analysis. Four participants did not report self-compassion data (across T6–T8) and were not included in the study. The final sample therefore included 264 older adults. Following the assessment of self-compassion, participants were assessed every 2 years for a total of 3 waves (2 years later: *n* = 226; 4 years later: *n* = 176). Study attrition was due to death (*n*= 17), lost contact (*n* = 24), refusing to participate (*n* = 36), sickness (*n* = 6), unable to follow directions (*n* = 3), personal reasons (*n* = 1), or unknown reasons (*n* = 1). Informed consent was obtained from all participants in the study prior to participation. The distribution of sociodemographic and health variables of the sample was within the normative range of older Canadians residing at home (National Advisory Council on Aging, 2006; see also Table 1).

**Table 1.** Means, standard deviations, frequencies and zero-order correlations of main study variables (*n* = 264).

Construct	Mean (SD) or %	1	2	3	4	5	6
Self-compassion (average)	41.30 (5.85)						
Chronic Illness (average)	2.59 (1.92)	−0.04					
Daily Physical Symptoms (average)	1.23 (1.28)	−0.15*	0.52**				
Age	75.25 (7.85)	0.02	0.26**	0.18**			
Female	60.2%	0.02	−0.01	0.17**	−0.05		
BMI^a^	27.10 (4.84)	−0.03	0.15*	0.08	−0.04	−0.08	
SES	−0.01 (0.79)	0.15*	−0.20**	−0.19**	−0.18**	−0.16**	−0.11

a *N* is slightly reduced for this construct due to missing data (*n* = 258).

*p ⩽ 0.05; ^**^p ⩽ 0.01.

### Materials

#### Self-compassion

Self-compassion was measured using the 12 item self-compassion scale at each wave (Raes et al., 2011). The scale asked participants to think about how they typically act during difficult times. Example items include: “When I fail at something important to me, I become consumed by feelings of inadequacy” or “I try to see my failings as part of the human condition.” Participants recorded their responses on a 5-point Likert type scale ranging from *almost never* = 0 to *almost always* = 4. A total self-compassion score was obtained for each wave by computing a sum score of the 12 items after negatively formulated items were reverse coded. Across T6–T8 the scale showed satisfactory reliability (α = 0.80–0.82). Self-compassion scores were significantly correlated across waves (*r*s > 0.70, *ps* < 0.001). Further, across all participants self-compassion generally increased over the course of the study (*coefficient* = 0.17, *SE* = 0.09, *T-ratio* = 2.00, *p* < 0.05). To obtain the most reliable measure of individual differences in self-compassion, we averaged self-compassion scores across all waves to compute an average measure of self-compassion across the study. Note that hypotheses-related significant effects, reported later, remained significant if we conducted analyses with only T6 scores of self-compassion (*n* = 261; three participants did not report self-compassion at T6).

#### Daily physical symptoms

At each wave participants completed a three-day daily survey that included a 12-item checklist of daily physical symptoms (e.g. stomach pain, headaches, constipation; Wrosch and Schulz, 2008). The number of symptoms was counted each day and averaged across the 3 days for an indicator of daily physical symptoms for each wave. During the 3 days of the first analyzed wave (i.e. T6), 32.6% of participants reported a daily average of zero physical symptoms, 28.0% between 0 and 1 symptoms, 19.3% between 1 and 2 symptoms, 8.7% between 2 and 3 symptoms, and 11.4% more than 3 symptoms.

#### Chronic illness

Chronic illness was assessed at each wave using a 17-item checklist of different chronic illnesses (e.g. cardiovascular problems, arthritis, diabetes, high blood pressure; Wrosch and Schulz, 2008). At each wave the number of chronic illnesses reported was counted to represent a total score of chronic illness. In the first analyzed wave, 10.6% had zero chronic illness, 19.7% had one chronic illness, 23.5% had two chronic illnesses, 22.0% had three chronic illnesses, 11.0% had four chronic illnesses, and 13.3% had 5 or more chronic illnesses.

#### Covariates

We included different sociodemographic and health-relevant covariates: sex, age, SES, and BMI. Sex was coded as 1 = Male and 2 = Female. SES was indexed using highest education completed, yearly family income, and perceived social status. The three standardized variables were significantly correlated (*r*s > 0.33; *p*s < 0.001) and were averaged together to create a composite SES variable. Research assistants objectively measured weight and height to calculate BMI.

### Data analyses

Preliminary analyses were conducted by calculating descriptive statistics and frequencies of the main study variables and their zero-order correlations (see Table 1). The main hypotheses were tested in two separate growth-curve models, predicting trajectories of daily physical symptoms and chronic illness over 4 years (using HLM 6.0, Raudenbush, 2004). The reported effects are based on models using restricted maximum likelihood estimation and robust standard errors. At Level 1 we estimated variance in participants’ daily physical symptoms and chronic illness across 4 years as function of an intercept, person-centered scores of time in the study and a residual term. In this case, the intercept represented participants’ average levels of daily physical symptoms and chronic illness across 4 years, while the time slope coefficient represents the yearly changes in daily physical symptoms and chronic illness from baseline to 4 years later.

At level 2 the intercept and slope of daily physical symptoms and chronic illness was predicted as a function of average self-compassion and covariates (sex, age, SES, BMI). All Level 2 main effect predictors were standardized prior to conducting the analyses. An interaction term between self-compassion and age was added in a second step of the models. Significant interaction effects were followed up by calculating simple slopes of the effects of self-compassion on changes in daily physical symptoms and chronic illness over time for those in early old age (−1 SD; 67.40 years) and advanced old age (+1 SD; 83.10 years). Since HLM is capable of handling missing data at Level 1 (i.e. daily physical symptoms and chronic illness), missing data in these variables were not replaced. There was a small amount of missing data of main predictors variables (BMI: *n* = 6), which were replaced with the sample mean (Tabachnick et al., 2007).

### Data sharing statement

A de-identified participant data set containing the main study variables (chronic illness, daily health symptoms, self-compassion, and covariates) among the study sample (*n* = 264) will be uploaded to the journal’s FigShare repository upon publication. A SPSS syntax file used to prepare the data set for analysis in HLM 6.0 has been uploaded as a Supplementary File. In addition, we have also uploaded the detailed HLM 6.0 output of all main analyses as a Supplementary File that allow for replication of the main analyses of this study.

## Results

### Preliminary analyses

Sample characteristics are displayed in Table 1. More than half the sample were female (60.2%). Participants were on average 75 years old (Range = 59–93). Participants had an average of 2.59 chronic illnesses, and 1.23 daily physical symptoms. The average BMI of the sample was 27.10. There was a diverse socioeconomic background among our sample, approximately 33.3% had an income less than $34,000 CAD, 22.7% had an income between $34,001–51,000 CAD, 18.2% had an income between $51,001–$85,000 CAD, and 16.7% had an income greater than $85,001 CAD (approximately 9% of the sample did not provide income information). Education was also diverse across our sample, 36.7% completed high school or less, 38.3% completed college or a bachelor’s degree, and 17.8% completed a master’s degree or above (approximately 7% of the sample did not provide education information). Participants reported an average perceived social status of 6.67 which is slightly above mid-range.

Zero-order correlations among the main study variables are reported in Table 2. Self-compassion was associated with less daily physical symptoms and higher SES. Having more chronic illnesses were associated with more daily physical symptoms, being older, a higher BMI, and lower SES. Having more daily physical symptoms was associated with being older, being female, and lower SES. Being older and female was associated lower SES.

**Table 2.** Results of growth-curve analysis predicting chronic illness and daily physical symptoms by self-compassion, age and covariates (*n* = 264).

	Chronic illness				Daily physical symptoms			
	Intercept (Average levels)		Slope (Time)		Intercept (Average levels)		Slope (Time)	
	Coefficient (SE)	T-Ratio	Coefficient (SE)	T-Ratio	Coefficient (SE)	T-Ratio	Coefficient (SE)	T-Ratio
Level 1 (*β*_0_; *β*_1_)^a^	2.58 (0.12)**	22.25	0.04 (0.04)	1.17	1.22 (0.08)**	15.58	0.01 (0.02)	0.46
Level 2: Main effects and covariates								
Self-Compassion	−0.03 (0.11)	−0.29	−0.01 (0.03)	−0.27	−0.17 (0.07)**	−2.53	−0.00(0.02)	−0.11
Age	0.45 (0.11)**	4.20	0.04 (0.03)	1.27	0.23 (0.07)**	3.27	0.03 (0.02)	1.55
Female	−0.00 (0.11)	−0.02	0.02 (0.04)	0.53	0.22 (0.07)**	3.11	−0.00 (0.02)	−0.23
BMI	0.27 (0.11)*	2.41	0.03 (0.03)	1.01	0.12 (0.08)	1.47	0.02 (0.02)	1.43
SES	−0.26 (0.13)*	−1.94	−0.04 (0.04)	−1.04	−0.12 (0.08)	−1.53	0.01 (0.02)	0.35
Level 2: Interaction effect								
SC X Age	−0.16 (0.11)	−1.40	−0.08 (0.03)*	−2.45	−0.15 (0.08)*	−1.93	0.03 (0.02)	1.28

a The first parameter (e.g. *β*_0_) estimated the intercept, which represents participants’ average chronic illness or daily physical symptoms across 4 years, and the second parameter (e.g. *β*_1_) estimated the slope, which represents the within-person associations between years in study from baseline to 4 years later and participants’ chronic illness and daily physical symptoms. The Level 1 model had 263 *df*s, the Level 2 models had 258 *df*s, and the interaction had 257 *dfs*.

SE = standard error.

*p ⩽ .05; ^**^p ⩽ .01.

### Predicting daily physical symptoms

The Level 1 model predicting daily physical symptoms showed a significant effect of the intercept, indicating that participants’ average daily physical symptoms across waves were significantly different from zero (see Table 2). The time slope of daily physical symptoms was not significantly different from zero, indicating that daily physical symptoms did not significantly change over 4 years across all participants. The results from the Level 1 model for daily physical symptoms showed significant variance around participants’ average intercept, *χ*^2^ = 1687.95, *df* = 231, *p* < 0.001, but not slope, *χ*^2^ = 265.82 *df* = 231, *p* = 0.057.

The Level 2 model predicted the observed variance in the intercept and time slope of daily physical symptoms as a function of self-compassion, age, and the covariates. Of the covariates, being female and older age significantly predicted higher average daily physical symptoms. No covariates significantly predicted the time slope of daily physical symptoms. However, self-compassion significantly predicted the intercept (but not the time slope) of daily physical symptoms. Participants with higher, as compared to lower, self-compassion scores reported lower average levels of daily physical symptoms. Self-compassion explained an additional 1.93% of the variance in average daily physical symptoms.

**Figure 1.**
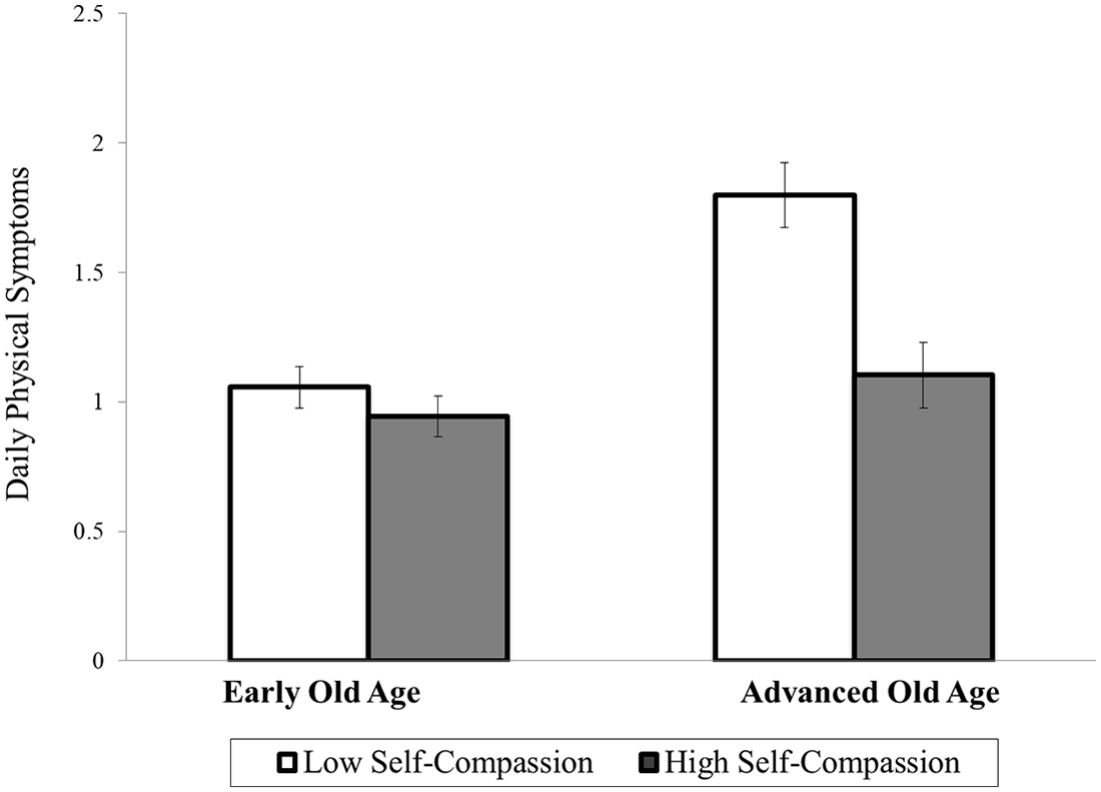
Average levels of daily physical symptoms across 4 years as a function of self-compassion and chronological age. Results for early versus advanced old age were plotted for 67.40 versus 83.10 years, respectively. Error bars represent the standard error.

In the next step we included an interaction term between self-compassion and age. The interaction term significantly predicted the intercept (but not the time slope) of daily physical symptoms. To illustrate the significant interaction, we used recommended growth-curve techniques (Preacher et al., 2006) and plotted the average levels of daily physical symptoms separately for those with low (−1 SD) and high (+1 SD) self-compassion in early (–1SD; 67.40 years) and advanced old age (+1 SD; 83.10 years) in Figure 1. The observed pattern suggests that being older was increasingly associated with more daily physical symptoms among participants with low self-compassion. In addition, the highest average levels of daily physical symptoms were observed among participants in advanced old age with lower self-compassion. Simple slope analyses supported this interpretation. Self-compassion significantly predicted average levels of daily physical symptoms among participants in advanced old age (*coefficient* = −0.35, *SE* = 0.13, *T-ratio* = −2.76, *p* < 0.01), but not among their counterparts in early old age (*coefficient* = −0.06, *SE* = 0.08, *T-ratio* = −0.71, *p* = 0.48). Including the interaction between self-compassion and age in the model explained an additional 1.13% of variance in average daily physical symptoms (controlling for main effects and all covariates).

### Predicting chronic illness

The Level 1 model predicting chronic illness showed a significant effect of the intercept, indicating the average chronic illness across all participants were significantly different from zero. The time slope of chronic illness was not significantly different from zero, suggesting that across all participants chronic illness did not change over time. Finally, the Level 1 model displayed significant variance around participants’ average intercept, *χ*^2^ = 1810.34, *df* = 231, *p* < 0.001, and slope, *χ*^2^ = 368.23, *df* = 231, *p* < 0.001.

The Level 2 model attempted to predict the variance in the intercept and slope of participants’ chronic illness as a function of self-compassion, age, and covariates. Of the covariates, higher BMI, lower SES, and being older was significantly associated with higher intercept values (i.e. average levels) of chronic illness across all waves. No other covariates or self-compassion significantly predicted the intercept or time slope.

**Figure 2.**
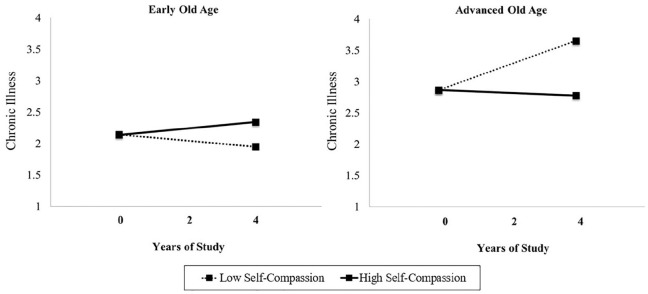
Changes in levels of chronic illness over 4 years as a function of self-compassion and chronological age. Results for early versus advanced old age were plotted for 67.40 versus 83.10 years, respectively.

For the final step of the analysis, we added the self-compassion and age interaction term to the model. The interaction term significantly predicted the time slope, but not the intercept, of chronic illness. To examine the interaction of self-compassion and age on the time slope of participants’ chronic illness, we plotted the trajectories of chronic illness across waves separately for participants with high (+1 SD) and low self-compassion (−1 SD) in early (−1 SD; 67.40 years) and advanced old age (+1 SD; 83.10 years). As depicted in Figure 2, levels of chronic illnesses were comparable at the beginning of the study period for young-old and older-old participants with high versus low self-compassion. However, simple slope analyses of the obtained interaction showed that chronic illness increased over 4 years among participants in advanced old age who reported low self-compassion (*coefficient* = 0.20, *SE* = 0.07, *T-ratio* = 2.79, *p* < 0.01). By contrast, chronic illness did not significantly increase among participants in advanced old age with high self-compassion (*coefficient* = −0.02, *SE* = 0.09, *T-ratio* = −0.23, *p* = 0.82). Among participants in early old age, chronic illness did not significantly change for those with either low (*coefficient* = −0.05, *SE* = 0.04, *T-ratio* = −1.13, *p* = 0.26) or high (*coefficient* = 0.05, *SE* = 0.06, *T-ratio* = 0.87, *p* = 0.39) self-compassion. The addition of the self-compassion by age interaction to the model explained additional 3.83% of variance in the time slope of participants’ chronic illness (controlling for main effects and all covariates).

## Discussion

The present study showed that self-compassion can predict levels and trajectories of daily physical symptoms and chronic health problems in older adulthood. More specifically, self-compassion was associated with fewer daily physical symptoms on average over 4 years across the entire sample. This result was moderated by age such that self-compassion predicted higher average levels of daily physical symptoms among people in advanced, but not early, old age. In addition, the results showed that lower, as compared to higher, self-compassion was associated with increases in chronic disease over 4 years among participants in advanced, but not early, old age. These age effects were independent of sociodemographic and health relevant covariates (sex, BMI, SES), and explained substantial variance in the age-related levels and longitudinal trajectories of daily physical symptoms and chronic health problems over time.

These results suggest that self-compassion is a beneficial psychological factor in promoting physical health among older adults. While the literature on self-compassion and psychological well-being is well established (Brown et al., 2019), the link between self-compassion and older adults’ physical health has been examined less and extant findings are largely cross-sectional (Phillips and Hine, 2019). This study therefore extends this literature by providing longitudinal evidence, documenting that self-compassion can not only relate to older adults’ physical health concurrently, but that it may also play a role in preventing development of physical health problems over time.

The study further supported our hypotheses by showing evidence for an enhanced importance of self-compassion for protecting physical health outcomes in advanced old age. Different from individuals in early old age, high, as compared with low, self-compassion was associated in advanced old age with generally lower levels of daily physical symptoms and fewer increases in the number of chronic illnesses. Although we predicted that self-compassion would also exert some health benefits in early old age, and increase its adaptive function during older adulthood, there was little evidence for differences in health as a function of self-compassion in early old age. One explanation for this pattern of findings is that other personality variables, which support counteracting stress-experiences, could play a more important role in early old age (e.g. optimism or goal engagement, Barlow et al., 2017; Wrosch et al., 2017).

Overall, the obtained findings are consistent with motivational theories of life-span development, which highlight that the adaptive value of certain self-regulation factors is age-dependent and becomes increasingly important in advanced old age (Heckhausen et al., 2019; Jobin and Wrosch, 2016). During old age, many individuals experience an increase in often irrevocable losses and uncontrollable stressors (Baltes and Smith, 2003; Heckhausen et al., 2019), which are likely to contribute to poor health outcomes over time (Barlow et al., 2017; Wrosch et al., 2006). As such, it is particularly important how older adults cope with an age-related shift in these life experiences. Older adults who experience a decline in their resources and opportunities to overcome pressing challenges may not be able to effectively address some of their problems, which could reduce the effectiveness of psychological mechanisms that support goal attainment (e.g. persistence or optimism; Wrosch et al., 2000, 2017). In such circumstances, the use of self-protective strategies that promote acceptance and disengagement from unattainable goals may be more beneficial (Jobin and Wrosch, 2016). Self-compassion represents such a psychological factor that has been shown to promote the use of self-protective strategies (Allen and Leary, 2010; Perez-Blasco et al., 2016). It may therefore increase its adaptive health-related function in advanced old age.

Note that the health benefits deriving from self-compassion could also accrue through other pathways. For example, self-compassion may not only promote adaptive coping, but could also facilitate positive health behaviors and reduced biological stress reactivity (Allen and Leary, 2010; Breines et al., 2014a, 2014b; Friis et al., 2015; Terry and Leary, 2011). In advanced old age, however, engagement in some health behaviors, such as physical activity, can become increasingly difficult (Bijnen et al., 1998). Consistent with this possibility, a meta-analysis has demonstrated that the association between self-compassion and health behaviors is reduced as individuals age (Phillips and Hine, 2019). As such, it seems more likely that self-compassion promotes physical health towards the end of life via adaptive coping and reduced stress reactivity. In fact, research on self-compassion interventions in older adult populations has demonstrated increases in self-protective coping strategies, such as positive reappraisal and a reduced negative self-focus (Perez-Blasco et al., 2016). As a consequence, self-compassion could help older adults to accept that certain problems can no longer be resolved and facilitate disengagement from unattainable goals. The psychological benefits of such self-regulation behaviors (Heckhausen et al., 2019; Wrosch and Scheier, 2020), in turn, could exert downstream consequences on the regulation of hormonal and immune processes that underlie the development of disease (e.g. cortisol or inflammation, Breines et al., 2014a, 2014b; Herriot et al., 2018; McEwen, 1998).

We further acknowledge that the predictive patterns observed for older adults’ daily physical symptoms and chronic illness were not identical. First, in advanced old age, self-compassion was cross-sectionally associated with fewer daily physical symptoms, but not chronic illnesses, across the 4-year study period. Second, self-compassion predicted fewer longitudinal increases in advanced old age only for chronic illness, but not for daily physical symptoms. To explain these differences in the patterns observed, it may be important to consider that daily physical symptoms may not only reflect manifest chronic illnesses, but can also represent early and sub-clinical signs of a developing disease that could take time before it manifests fully or is diagnosed (Wrosch and Schulz, 2008). With these considerations in mind, it would be possible that individual differences in self-compassion had an earlier effect on older participants’ daily physical symptoms, which plateaued during the observed study period. Further, such earlier and high levels of daily physical symptoms could have contributed to increasing levels of chronic disease during the observed study period, among participants in advanced old age with low self-compassion. This possibility could explain the observed chronically elevated levels of daily health symptoms among individuals in advanced old age with low self-compassion, and their steadily increasing levels of chronic illness.

We also acknowledge that the percentage of variance explained in health outcomes by individual differences in self-compassion was relatively small (1.13–3.83%). However, we would like to note that the difference between low versus high self-compassion in advanced old age translated into one daily physical symptom across the entire study period (see Figure 1) and one additional chronic illness after 4 years of study (see Figure 2). Chronic illnesses, such as cardiovascular problems, arthritis, or diabetes, represent pressing health problems and can induce significant distress and disability that can impair an individual’s quality of life (Freund and Baltes, 2000; Williamson and Schulz, 1995; Wrosch and Schulz, 2008). We therefore feel that although the amount of explained variance is relatively small, it could be clinically relevant. In addition, we would like to note that small effects may be a result of the relatively short follow-up of 4 years. As a result, it would be possible that these effects could be larger when examined over longer periods of time (e.g. 10 years) which could provide stronger clinical relevance for supporting health in old age.

Overall the reported findings have important implications for theories of successful aging. They bolster life-span developmental theories and research, which posit that the function of self-protective psychological factors is age-dependent and becomes increasingly important in advanced old age (Heckhausen et al., 2019; Jobin and Wrosch, 2016). To this end, our study highlights that a modifiable psychological variable, self-compassion, may be beneficial for both daily and chronic health outcomes as older adults advance in age. Given that daily and chronic illness can further jeopardize older adults’ quality of life by eliciting additional distress and causing disability (Freund and Baltes, 2000), our findings point to the importance of fostering adaptive self-regulation factors, like self-compassion, particularly among individuals in advanced old age. Indeed, there is accumulating evidence that older adults experience sharp declines across different areas of life, including physical health, as they approach the end of their lives (Baltes and Smith, 2003; Gerstorf et al., 2013). However, little is known about the psychological factors that could slow down or prevent such losses. To this end, our findings could inform much needed intervention research to improve quality of life in aging populations and reduce exorbitant health care spending currently dedicated to managing daily physical symptoms and chronic health conditions.

While there are many strengths of this study, such as the use of a longitudinal design and a community dwelling older adult sample, there are also some limitations that should be addressed in future research. First, the sample size is relatively small and may not be generalizable to all older adults. Therefore, research should aim to replicate these findings in larger and more diverse samples. Second, we acknowledge that this study focused on self-compassion as a predictor of physical health, and did not examine other broader, potentially protective personality constructs. Since individual difference variables, such as emotional stability, could also contribute to maintaining health in old age (Lahey, 2009), future research should examine a wider array of personality variables. Third, our analysis did not address the mechanisms by which self-compassion promotes physical health. It will be important for future research to illuminate how self-compassion could promote better health over time. For example, it would be interesting to examine whether certain behaviors in response to stress experiences (e.g. coping or health behaviors) and accruing emotional states could explain the documented associations between self-compassion and physical health outcomes. In addition, changes in biological markers of developing disease (e.g. cortisol or chronic inflammation) could mediate the relations between self-compassion and older adults’ daily and chronic health problems. Fourth, although our study controlled for a number of health-relevant covariates (e.g. sex, BMI, or SES), there are additional factors that were not included in our study, but could influence physical health. For example, differences in race or ethnicity may play and important role (Mays et al., 2007). From our perspective, it may be the most vulnerable segments of the population that could benefit from self-compassion in old age. Finally, the correlational design of our study cannot determine causality in the association between self-compassion and physical health. Given that self-compassion is a modifiable psychological variable (e.g. Friis et al., 2016; Neff and Germer, 2013), we think that there is merit for intervention studies to conduct research to help elucidate the casual pathways linking self-compassion and physical health in old age.

## Conclusions

The present study highlights self-compassion as an important psychological factor for older adults’ physical health over time. By comparing people in early and advanced old age, the study’s results suggest that self-compassion becomes important for protecting daily and chronic health outcomes particularly in advanced old age. These results add to motivational theories of lifespan development and successful aging and have implications for interventions that promote self-compassion as a way to improve the health of the aging population.

## Supplemental Material

sj-docx-2-hpq-10.1177_13591053211002326 – Supplemental material for Self-compassion as predictor of daily physical symptoms and chronic illness across older adulthood Supplemental material, sj-docx-2-hpq-10.1177_13591053211002326 for Self-compassion as predictor of daily physical symptoms and chronic illness across older adulthood by Heather Herriot and Carsten Wrosch in Journal of Health Psychology

sj-docx-3-hpq-10.1177_13591053211002326 – Supplemental material for Self-compassion as predictor of daily physical symptoms and chronic illness across older adulthood Supplemental material, sj-docx-3-hpq-10.1177_13591053211002326 for Self-compassion as predictor of daily physical symptoms and chronic illness across older adulthood by Heather Herriot and Carsten Wrosch in Journal of Health Psychology

sj-sav-1-hpq-10.1177_13591053211002326 – for Self-compassion as predictor of daily physical symptoms and chronic illness across older adulthood sj-sav-1-hpq-10.1177_13591053211002326 for Self-compassion as predictor of daily physical symptoms and chronic illness across older adulthood by Heather Herriot and Carsten Wrosch in Journal of Health Psychology This article is distributed under the terms of the Creative Commons Attribution 4.0 License ( http://www.creativecommons.org/licenses/by/4.0/) which permits any use, reproduction and distribution of the work without further permission provided the original work is attributed as specified on the SAGE and Open Access pages ( https://us.sagepub.com/en-us/nam/open-access-at-sage).

sj-sps-4-hpq-10.1177_13591053211002326 – Supplemental material for Self-compassion as predictor of daily physical symptoms and chronic illness across older adulthood Supplemental material, sj-sps-4-hpq-10.1177_13591053211002326 for Self-compassion as predictor of daily physical symptoms and chronic illness across older adulthood by Heather Herriot and Carsten Wrosch in Journal of Health Psychology
