# Supplementary material for: Self-compassion as predictor of daily physical symptoms and chronic illness across older adulthood
Source: J Health Psychol. 2021 Mar 27;27(7):1697–709. doi: 10.1177/13591053211002326 (PMC9092919; doi:10.1177/13591053211002326)
Supplement: sj-docx-2-hpq-10.1177_13591053211002326 – Supplemental material for Self-compassion as predictor of daily physical symptoms and chronic illness across older adulthood [file sj-docx-2-hpq-10.1177_13591053211002326.docx]

**Explanatory Memo**

All materials necessary to replicate the main analyses of this study are provided. First, a data file containing all main study variables (chronic illness, daily health symptoms, self-compassion, age, sex, BMI, and SES) of the study sample (N = 264) are included using SPSS 25.0. Participant ID information was removed to ensure confidentiality. Second, a syntax file used to prepare the Level 1 and Level 2 data files for the main analyses in HLM has been provided. Third, all outputs of the main analyses in HLM have been provided. The outputs provide sufficient information to understand each model conducted as well as the detailed output of each analysis. All analyses were conducted on HLM 6.0. Additional description of the analyses can be found in the Data Analyses section of the Methods in the manuscript.
