# Supplementary material for: Self-compassion as predictor of daily physical symptoms and chronic illness across older adulthood
Source: J Health Psychol. 2021 Mar 27;27(7):1697–709. doi: 10.1177/13591053211002326 (PMC9092919; doi:10.1177/13591053211002326)
Supplement: sj-docx-3-hpq-10.1177_13591053211002326 – Supplemental material for Self-compassion as predictor of daily physical symptoms and chronic illness across older adulthood [file sj-docx-3-hpq-10.1177_13591053211002326.docx]

**Chronic Illness Model**

**Level 1 Output**

Program: HLM 6 Hierarchical Linear and Nonlinear Modeling

Authors: Stephen Raudenbush, Tony Bryk, & Richard Congdon

Publisher: Scientific Software International, Inc. (c) 2000

techsupport@ssicentral.com

www.ssicentral.com

-------------------------------------------------------------------------------

Module: HLM2.EXE (6.08.29257.1)

Date: 30 June 2020, Tuesday

Time: 11: 5:42

-------------------------------------------------------------------------------

SPECIFICATIONS FOR THIS HLM2 RUN

Problem Title: no title

The data source for this run = SC_Health.mdm

The command file for this run = C:\Users\heath\AppData\Local\Temp\whlmtemp.hlm

Output file name = C:\Users\heath\Dropbox\hlm2.txt

The maximum number of level-1 units = 792

The maximum number of level-2 units = 264

The maximum number of iterations = 100

Method of estimation: restricted maximum likelihood

Weighting Specification

-----------------------

Weight

Variable

Weighting? Name Normalized?

Level 1 no

Level 2 no

Precision no

The outcome variable is HILL

The model specified for the fixed effects was:

----------------------------------------------------

Level-1 Level-2

Coefficients Predictors

---------------------- ---------------

INTRCPT1, P0 INTRCPT2, B00

* TIME slope, P1 INTRCPT2, B10

'*' - This level-1 predictor has been centered around its group mean.

The model specified for the covariance components was:

---------------------------------------------------------

Sigma squared (constant across level-2 units)

Tau dimensions

INTRCPT1

TIME slope

Summary of the model specified (in equation format)

---------------------------------------------------

Level-1 Model

Y = P0 + P1*(TIME) + E

Level-2 Model

P0 = B00 + R0

P1 = B10 + R1

Run-time deletion has reduced the number of level-1 records to 666

Iterations stopped due to small change in likelihood function

******* ITERATION 26 *******

Sigma_squared = 1.12352

Tau

INTRCPT1,P0 3.07759 0.25470

TIME,P1 0.25470 0.10909

Tau (as correlations)

INTRCPT1,P0 1.000 0.440

TIME,P1 0.440 1.000

----------------------------------------------------

Random level-1 coefficient Reliability estimate

----------------------------------------------------

INTRCPT1, P0 0.879

TIME, P1 0.371

----------------------------------------------------

Note: The reliability estimates reported above are based on only 232 of 264

units that had sufficient data for computation. Fixed effects and variance

components are based on all the data.

The value of the likelihood function at iteration 26 = -1.302241E+003

The outcome variable is HILL

Final estimation of fixed effects:

----------------------------------------------------------------------------

Standard Approx.

Fixed Effect Coefficient Error T-ratio d.f. P-value

----------------------------------------------------------------------------

For INTRCPT1, P0

INTRCPT2, B00 2.583817 0.116324 22.212 263 0.000

For TIME slope, P1

INTRCPT2, B10 0.041311 0.035372 1.168 263 0.244

----------------------------------------------------------------------------

The outcome variable is HILL

Final estimation of fixed effects

(with robust standard errors)

----------------------------------------------------------------------------

Standard Approx.

Fixed Effect Coefficient Error T-ratio d.f. P-value

----------------------------------------------------------------------------

For INTRCPT1, P0

INTRCPT2, B00 2.583817 0.116106 22.254 263 0.000

For TIME slope, P1

INTRCPT2, B10 0.041311 0.035305 1.170 263 0.243

----------------------------------------------------------------------------

Final estimation of variance components:

-----------------------------------------------------------------------------

Random Effect Standard Variance df Chi-square P-value

Deviation Component

-----------------------------------------------------------------------------

INTRCPT1, R0 1.75431 3.07759 231 1810.33534 0.000

TIME slope, R1 0.33028 0.10909 231 368.22801 0.000

level-1, E 1.05996 1.12352

-----------------------------------------------------------------------------

Note: The chi-square statistics reported above are based on only 232 of 264

units that had sufficient data for computation. Fixed effects and variance

components are based on all the data.

Statistics for current covariance components model

--------------------------------------------------

Deviance = 2604.482468

Number of estimated parameters = 4

**Main Effects Output**

Program: HLM 6 Hierarchical Linear and Nonlinear Modeling

Authors: Stephen Raudenbush, Tony Bryk, & Richard Congdon

Publisher: Scientific Software International, Inc. (c) 2000

techsupport@ssicentral.com

www.ssicentral.com

-------------------------------------------------------------------------------

Module: HLM2.EXE (6.08.29257.1)

Date: 30 June 2020, Tuesday

Time: 11:10: 2

-------------------------------------------------------------------------------

SPECIFICATIONS FOR THIS HLM2 RUN

Problem Title: no title

The data source for this run = SC_Health.mdm

The command file for this run = C:\Users\heath\AppData\Local\Temp\whlmtemp.hlm

Output file name = C:\Users\heath\Dropbox\hlm2.txt

The maximum number of level-1 units = 792

The maximum number of level-2 units = 264

The maximum number of iterations = 100

Method of estimation: restricted maximum likelihood

Weighting Specification

-----------------------

Weight

Variable

Weighting? Name Normalized?

Level 1 no

Level 2 no

Precision no

The outcome variable is HILL

The model specified for the fixed effects was:

----------------------------------------------------

Level-1 Level-2

Coefficients Predictors

---------------------- ---------------

INTRCPT1, P0 INTRCPT2, B00

ZAGE_6, B01

ZSEX_6, B02

ZSES_M6, B03

ZAVGSC, B04

ZBMI_O6_, B05

* TIME slope, P1 INTRCPT2, B10

ZAGE_6, B11

ZSEX_6, B12

ZSES_M6, B13

ZAVGSC, B14

ZBMI_O6_, B15

'*' - This level-1 predictor has been centered around its group mean.

The model specified for the covariance components was:

---------------------------------------------------------

Sigma squared (constant across level-2 units)

Tau dimensions

INTRCPT1

TIME slope

Summary of the model specified (in equation format)

---------------------------------------------------

Level-1 Model

Y = P0 + P1*(TIME) + E

Level-2 Model

P0 = B00 + B01*(ZAGE_6) + B02*(ZSEX_6) + B03*(ZSES_M6) + B04*(ZAVGSC)

+ B05*(ZBMI_O6_) + R0

P1 = B10 + B11*(ZAGE_6) + B12*(ZSEX_6) + B13*(ZSES_M6) + B14*(ZAVGSC)

+ B15*(ZBMI_O6_) + R1

Run-time deletion has reduced the number of level-1 records to 666

Iterations stopped due to small change in likelihood function

******* ITERATION 32 *******

Sigma_squared = 1.12130

Tau

INTRCPT1,P0 2.73450 0.21240

TIME,P1 0.21240 0.11063

Tau (as correlations)

INTRCPT1,P0 1.000 0.386

TIME,P1 0.386 1.000

----------------------------------------------------

Random level-1 coefficient Reliability estimate

----------------------------------------------------

INTRCPT1, P0 0.866

TIME, P1 0.374

----------------------------------------------------

Note: The reliability estimates reported above are based on only 232 of 264

units that had sufficient data for computation. Fixed effects and variance

components are based on all the data.

The value of the likelihood function at iteration 32 = -1.304551E+003

The outcome variable is HILL

Final estimation of fixed effects:

----------------------------------------------------------------------------

Standard Approx.

Fixed Effect Coefficient Error T-ratio d.f. P-value

----------------------------------------------------------------------------

For INTRCPT1, P0

INTRCPT2, B00 2.589336 0.110572 23.418 258 0.000

ZAGE_6, B01 0.453698 0.113888 3.984 258 0.000

ZSEX_6, B02 -0.002108 0.113283 -0.019 258 0.985

ZSES_M6, B03 -0.257713 0.116825 -2.206 258 0.028

ZAVGSC, B04 -0.031128 0.111982 -0.278 258 0.781

ZBMI_O6_, B05 0.273540 0.111816 2.446 258 0.015

For TIME slope, P1

INTRCPT2, B10 0.043708 0.035924 1.217 258 0.225

ZAGE_6, B11 0.044319 0.037298 1.188 258 0.236

ZSEX_6, B12 0.019093 0.036461 0.524 258 0.601

ZSES_M6, B13 -0.037969 0.037361 -1.016 258 0.311

ZAVGSC, B14 -0.008739 0.035332 -0.247 258 0.805

ZBMI_O6_, B15 0.033282 0.034795 0.957 258 0.340

----------------------------------------------------------------------------

The outcome variable is HILL

Final estimation of fixed effects

(with robust standard errors)

----------------------------------------------------------------------------

Standard Approx.

Fixed Effect Coefficient Error T-ratio d.f. P-value

----------------------------------------------------------------------------

For INTRCPT1, P0

INTRCPT2, B00 2.589336 0.110010 23.537 258 0.000

ZAGE_6, B01 0.453698 0.108123 4.196 258 0.000

ZSEX_6, B02 -0.002108 0.114257 -0.018 258 0.985

ZSES_M6, B03 -0.257713 0.133048 -1.937 258 0.053

ZAVGSC, B04 -0.031128 0.107685 -0.289 258 0.773

ZBMI_O6_, B05 0.273540 0.113674 2.406 258 0.017

For TIME slope, P1

INTRCPT2, B10 0.043708 0.037699 1.159 258 0.248

ZAGE_6, B11 0.044319 0.034947 1.268 258 0.206

ZSEX_6, B12 0.019093 0.036139 0.528 258 0.597

ZSES_M6, B13 -0.037969 0.036694 -1.035 258 0.302

ZAVGSC, B14 -0.008739 0.032284 -0.271 258 0.787

ZBMI_O6_, B15 0.033282 0.032940 1.010 258 0.314

----------------------------------------------------------------------------

Final estimation of variance components:

-----------------------------------------------------------------------------

Random Effect Standard Variance df Chi-square P-value

Deviation Component

-----------------------------------------------------------------------------

INTRCPT1, R0 1.65363 2.73450 226 1603.82334 0.000

TIME slope, R1 0.33261 0.11063 226 362.90948 0.000

level-1, E 1.05891 1.12130

-----------------------------------------------------------------------------

Note: The chi-square statistics reported above are based on only 232 of 264

units that had sufficient data for computation. Fixed effects and variance

components are based on all the data.

Statistics for current covariance components model

--------------------------------------------------

Deviance = 2609.101629

Number of estimated parameters = 4

**Interaction Output**

Program: HLM 6 Hierarchical Linear and Nonlinear Modeling

Authors: Stephen Raudenbush, Tony Bryk, & Richard Congdon

Publisher: Scientific Software International, Inc. (c) 2000

techsupport@ssicentral.com

www.ssicentral.com

-------------------------------------------------------------------------------

Module: HLM2.EXE (6.08.29257.1)

Date: 30 June 2020, Tuesday

Time: 11:17:34

-------------------------------------------------------------------------------

SPECIFICATIONS FOR THIS HLM2 RUN

Problem Title: no title

The data source for this run = SC_Health.mdm

The command file for this run = C:\Users\heath\AppData\Local\Temp\whlmtemp.hlm

Output file name = C:\Users\heath\Dropbox\hlm2.txt

The maximum number of level-1 units = 792

The maximum number of level-2 units = 264

The maximum number of iterations = 100

Method of estimation: restricted maximum likelihood

Weighting Specification

-----------------------

Weight

Variable

Weighting? Name Normalized?

Level 1 no

Level 2 no

Precision no

The outcome variable is HILL

The model specified for the fixed effects was:

----------------------------------------------------

Level-1 Level-2

Coefficients Predictors

---------------------- ---------------

INTRCPT1, P0 INTRCPT2, B00

ZAGE_6, B01

ZSEX_6, B02

ZSES_M6, B03

ZAVGSC, B04

ZBMI_O6_, B05

AGEX8SC, B06

* TIME slope, P1 INTRCPT2, B10

ZAGE_6, B11

ZSEX_6, B12

ZSES_M6, B13

ZAVGSC, B14

ZBMI_O6_, B15

AGEX8SC, B16

'*' - This level-1 predictor has been centered around its group mean.

The model specified for the covariance components was:

---------------------------------------------------------

Sigma squared (constant across level-2 units)

Tau dimensions

INTRCPT1

TIME slope

Summary of the model specified (in equation format)

---------------------------------------------------

Level-1 Model

Y = P0 + P1*(TIME) + E

Level-2 Model

P0 = B00 + B01*(ZAGE_6) + B02*(ZSEX_6) + B03*(ZSES_M6) + B04*(ZAVGSC)

+ B05*(ZBMI_O6_) + B06*(AGEX8SC) + R0

P1 = B10 + B11*(ZAGE_6) + B12*(ZSEX_6) + B13*(ZSES_M6) + B14*(ZAVGSC)

+ B15*(ZBMI_O6_) + B16*(AGEX8SC) + R1

Run-time deletion has reduced the number of level-1 records to 666

Iterations stopped due to small change in likelihood function

******* ITERATION 31 *******

Sigma_squared = 1.11978

Tau

INTRCPT1,P0 2.72478 0.20051

TIME,P1 0.20051 0.10639

Tau (as correlations)

INTRCPT1,P0 1.000 0.372

TIME,P1 0.372 1.000

----------------------------------------------------

Random level-1 coefficient Reliability estimate

----------------------------------------------------

INTRCPT1, P0 0.866

TIME, P1 0.366

----------------------------------------------------

Note: The reliability estimates reported above are based on only 232 of 264

units that had sufficient data for computation. Fixed effects and variance

components are based on all the data.

The value of the likelihood function at iteration 31 = -1.305451E+003

The outcome variable is HILL

Final estimation of fixed effects:

----------------------------------------------------------------------------

Standard Approx.

Fixed Effect Coefficient Error T-ratio d.f. P-value

----------------------------------------------------------------------------

For INTRCPT1, P0

INTRCPT2, B00 2.592411 0.110420 23.478 257 0.000

ZAGE_6, B01 0.447602 0.113794 3.933 257 0.000

ZSEX_6, B02 -0.009386 0.113227 -0.083 257 0.934

ZSES_M6, B03 -0.253042 0.116687 -2.169 257 0.031

ZAVGSC, B04 -0.059814 0.113762 -0.526 257 0.599

ZBMI_O6_, B05 0.273637 0.111637 2.451 257 0.015

AGEX8SC, B06 -0.157507 0.115072 -1.369 257 0.172

For TIME slope, P1

INTRCPT2, B10 0.044565 0.035648 1.250 257 0.213

ZAGE_6, B11 0.043014 0.037011 1.162 257 0.247

ZSEX_6, B12 0.013026 0.036295 0.359 257 0.720

ZSES_M6, B13 -0.036056 0.037072 -0.973 257 0.332

ZAVGSC, B14 -0.029734 0.036525 -0.814 257 0.416

ZBMI_O6_, B15 0.035312 0.034529 1.023 257 0.308

AGEX8SC, B16 -0.079348 0.036961 -2.147 257 0.033

----------------------------------------------------------------------------

The outcome variable is HILL

Final estimation of fixed effects

(with robust standard errors)

----------------------------------------------------------------------------

Standard Approx.

Fixed Effect Coefficient Error T-ratio d.f. P-value

----------------------------------------------------------------------------

For INTRCPT1, P0

INTRCPT2, B00 2.592411 0.109862 23.597 257 0.000

ZAGE_6, B01 0.447602 0.107407 4.167 257 0.000

ZSEX_6, B02 -0.009386 0.113751 -0.083 257 0.935

ZSES_M6, B03 -0.253042 0.133057 -1.902 257 0.058

ZAVGSC, B04 -0.059814 0.110821 -0.540 257 0.589

ZBMI_O6_, B05 0.273637 0.113043 2.421 257 0.016

AGEX8SC, B06 -0.157507 0.112936 -1.395 257 0.164

For TIME slope, P1

INTRCPT2, B10 0.044565 0.037163 1.199 257 0.232

ZAGE_6, B11 0.043014 0.034300 1.254 257 0.211

ZSEX_6, B12 0.013026 0.034961 0.373 257 0.709

ZSES_M6, B13 -0.036056 0.036386 -0.991 257 0.323

ZAVGSC, B14 -0.029734 0.034781 -0.855 257 0.394

ZBMI_O6_, B15 0.035312 0.032494 1.087 257 0.279

AGEX8SC, B16 -0.079348 0.032385 -2.450 257 0.015

----------------------------------------------------------------------------

Final estimation of variance components:

-----------------------------------------------------------------------------

Random Effect Standard Variance df Chi-square P-value

Deviation Component

-----------------------------------------------------------------------------

INTRCPT1, R0 1.65069 2.72478 225 1595.67239 0.000

TIME slope, R1 0.32618 0.10639 225 356.13497 0.000

level-1, E 1.05820 1.11978

-----------------------------------------------------------------------------

Note: The chi-square statistics reported above are based on only 232 of 264

units that had sufficient data for computation. Fixed effects and variance

components are based on all the data.

Statistics for current covariance components model

--------------------------------------------------

Deviance = 2610.901954

Number of estimated parameters = 4

**Daily Health Symptoms Model**

**Level 1 Output**

Program: HLM 6 Hierarchical Linear and Nonlinear Modeling

Authors: Stephen Raudenbush, Tony Bryk, & Richard Congdon

Publisher: Scientific Software International, Inc. (c) 2000

techsupport@ssicentral.com

www.ssicentral.com

-------------------------------------------------------------------------------

Module: HLM2.EXE (6.08.29257.1)

Date: 30 June 2020, Tuesday

Time: 11:29:29

-------------------------------------------------------------------------------

SPECIFICATIONS FOR THIS HLM2 RUN

Problem Title: no title

The data source for this run = SC_Health.mdm

The command file for this run = C:\Users\heath\AppData\Local\Temp\whlmtemp.hlm

Output file name = C:\Users\heath\Dropbox\hlm2.txt

The maximum number of level-1 units = 792

The maximum number of level-2 units = 264

The maximum number of iterations = 100

Method of estimation: restricted maximum likelihood

Weighting Specification

-----------------------

Weight

Variable

Weighting? Name Normalized?

Level 1 no

Level 2 no

Precision no

The outcome variable is DHSYMP

The model specified for the fixed effects was:

----------------------------------------------------

Level-1 Level-2

Coefficients Predictors

---------------------- ---------------

INTRCPT1, P0 INTRCPT2, B00

* TIME slope, P1 INTRCPT2, B10

'*' - This level-1 predictor has been centered around its group mean.

The model specified for the covariance components was:

---------------------------------------------------------

Sigma squared (constant across level-2 units)

Tau dimensions

INTRCPT1

TIME slope

Summary of the model specified (in equation format)

---------------------------------------------------

Level-1 Model

Y = P0 + P1*(TIME) + E

Level-2 Model

P0 = B00 + R0

P1 = B10 + R1

Run-time deletion has reduced the number of level-1 records to 666

Iterations stopped due to small change in likelihood function

******* ITERATION 530 *******

Sigma_squared = 0.57481

Tau

INTRCPT1,P0 1.37200 0.01889

TIME,P1 0.01889 0.00678

Tau (as correlations)

INTRCPT1,P0 1.000 0.196

TIME,P1 0.196 1.000

----------------------------------------------------

Random level-1 coefficient Reliability estimate

----------------------------------------------------

INTRCPT1, P0 0.864

TIME, P1 0.071

----------------------------------------------------

Note: The reliability estimates reported above are based on only 232 of 264

units that had sufficient data for computation. Fixed effects and variance

components are based on all the data.

The value of the likelihood function at iteration 530 = -1.024829E+003

The outcome variable is DHSYMP

Final estimation of fixed effects:

----------------------------------------------------------------------------

Standard Approx.

Fixed Effect Coefficient Error T-ratio d.f. P-value

----------------------------------------------------------------------------

For INTRCPT1, P0

INTRCPT2, B00 1.220081 0.078441 15.554 263 0.000

For TIME slope, P1

INTRCPT2, B10 0.009334 0.020286 0.460 263 0.645

----------------------------------------------------------------------------

The outcome variable is DHSYMP

Final estimation of fixed effects

(with robust standard errors)

----------------------------------------------------------------------------

Standard Approx.

Fixed Effect Coefficient Error T-ratio d.f. P-value

----------------------------------------------------------------------------

For INTRCPT1, P0

INTRCPT2, B00 1.220081 0.078291 15.584 263 0.000

For TIME slope, P1

INTRCPT2, B10 0.009334 0.020189 0.462 263 0.644

----------------------------------------------------------------------------

Final estimation of variance components:

-----------------------------------------------------------------------------

Random Effect Standard Variance df Chi-square P-value

Deviation Component

-----------------------------------------------------------------------------

INTRCPT1, R0 1.17133 1.37200 231 1687.95156 0.000

TIME slope, R1 0.08236 0.00678 231 265.82174 0.057

level-1, E 0.75816 0.57481

-----------------------------------------------------------------------------

Note: The chi-square statistics reported above are based on only 232 of 264

units that had sufficient data for computation. Fixed effects and variance

components are based on all the data.

Statistics for current covariance components model

--------------------------------------------------

Deviance = 2049.657367

Number of estimated parameters = 4

**Main Effects Output**

Program: HLM 6 Hierarchical Linear and Nonlinear Modeling

Authors: Stephen Raudenbush, Tony Bryk, & Richard Congdon

Publisher: Scientific Software International, Inc. (c) 2000

techsupport@ssicentral.com

www.ssicentral.com

-------------------------------------------------------------------------------

Module: HLM2.EXE (6.08.29257.1)

Date: 30 June 2020, Tuesday

Time: 11:31:58

-------------------------------------------------------------------------------

SPECIFICATIONS FOR THIS HLM2 RUN

Problem Title: no title

The data source for this run = SC_Health.mdm

The command file for this run = C:\Users\heath\AppData\Local\Temp\whlmtemp.hlm

Output file name = C:\Users\heath\Dropbox\hlm2.txt

The maximum number of level-1 units = 792

The maximum number of level-2 units = 264

The maximum number of iterations = 100

Method of estimation: restricted maximum likelihood

Weighting Specification

-----------------------

Weight

Variable

Weighting? Name Normalized?

Level 1 no

Level 2 no

Precision no

The outcome variable is DHSYMP

The model specified for the fixed effects was:

----------------------------------------------------

Level-1 Level-2

Coefficients Predictors

---------------------- ---------------

INTRCPT1, P0 INTRCPT2, B00

ZAGE_6, B01

ZSEX_6, B02

ZSES_M6, B03

ZAVGSC, B04

ZBMI_O6_, B05

* TIME slope, P1 INTRCPT2, B10

ZAGE_6, B11

ZSEX_6, B12

ZSES_M6, B13

ZAVGSC, B14

ZBMI_O6_, B15

'*' - This level-1 predictor has been centered around its group mean.

The model specified for the covariance components was:

---------------------------------------------------------

Sigma squared (constant across level-2 units)

Tau dimensions

INTRCPT1

TIME slope

Summary of the model specified (in equation format)

---------------------------------------------------

Level-1 Model

Y = P0 + P1*(TIME) + E

Level-2 Model

P0 = B00 + B01*(ZAGE_6) + B02*(ZSEX_6) + B03*(ZSES_M6) + B04*(ZAVGSC)

+ B05*(ZBMI_O6_) + R0

P1 = B10 + B11*(ZAGE_6) + B12*(ZSEX_6) + B13*(ZSES_M6) + B14*(ZAVGSC)

+ B15*(ZBMI_O6_) + R1

Run-time deletion has reduced the number of level-1 records to 666

Iterations stopped due to small change in likelihood function

******* ITERATION 390 *******

Sigma_squared = 0.57681

Tau

INTRCPT1,P0 1.22248 0.00975

TIME,P1 0.00975 0.00705

Tau (as correlations)

INTRCPT1,P0 1.000 0.105

TIME,P1 0.105 1.000

----------------------------------------------------

Random level-1 coefficient Reliability estimate

----------------------------------------------------

INTRCPT1, P0 0.849

TIME, P1 0.073

----------------------------------------------------

Note: The reliability estimates reported above are based on only 232 of 264

units that had sufficient data for computation. Fixed effects and variance

components are based on all the data.

The value of the likelihood function at iteration 390 = -1.031557E+003

The outcome variable is DHSYMP

Final estimation of fixed effects:

----------------------------------------------------------------------------

Standard Approx.

Fixed Effect Coefficient Error T-ratio d.f. P-value

----------------------------------------------------------------------------

For INTRCPT1, P0

INTRCPT2, B00 1.222551 0.074760 16.353 258 0.000

ZAGE_6, B01 0.230665 0.077031 2.994 258 0.003

ZSEX_6, B02 0.219978 0.076593 2.872 258 0.005

ZSES_M6, B03 -0.115089 0.078982 -1.457 258 0.146

ZAVGSC, B04 -0.174961 0.075689 -2.312 258 0.022

ZBMI_O6_, B05 0.115465 0.075564 1.528 258 0.128

For TIME slope, P1

INTRCPT2, B10 0.011589 0.020660 0.561 258 0.575

ZAGE_6, B11 0.029110 0.021393 1.361 258 0.175

ZSEX_6, B12 -0.004645 0.020899 -0.222 258 0.824

ZSES_M6, B13 0.008101 0.021428 0.378 258 0.705

ZAVGSC, B14 -0.002287 0.020190 -0.113 258 0.910

ZBMI_O6_, B15 0.024559 0.019830 1.238 258 0.217

----------------------------------------------------------------------------

The outcome variable is DHSYMP

Final estimation of fixed effects

(with robust standard errors)

----------------------------------------------------------------------------

Standard Approx.

Fixed Effect Coefficient Error T-ratio d.f. P-value

----------------------------------------------------------------------------

For INTRCPT1, P0

INTRCPT2, B00 1.222551 0.074377 16.437 258 0.000

ZAGE_6, B01 0.230665 0.070459 3.274 258 0.002

ZSEX_6, B02 0.219978 0.070645 3.114 258 0.002

ZSES_M6, B03 -0.115089 0.075169 -1.531 258 0.127

ZAVGSC, B04 -0.174961 0.069164 -2.530 258 0.012

ZBMI_O6_, B05 0.115465 0.078372 1.473 258 0.142

For TIME slope, P1

INTRCPT2, B10 0.011589 0.020482 0.566 258 0.572

ZAGE_6, B11 0.029110 0.018782 1.550 258 0.122

ZSEX_6, B12 -0.004645 0.020375 -0.228 258 0.820

ZSES_M6, B13 0.008101 0.023033 0.352 258 0.725

ZAVGSC, B14 -0.002287 0.021609 -0.106 258 0.916

ZBMI_O6_, B15 0.024559 0.017199 1.428 258 0.155

----------------------------------------------------------------------------

Final estimation of variance components:

-----------------------------------------------------------------------------

Random Effect Standard Variance df Chi-square P-value

Deviation Component

-----------------------------------------------------------------------------

INTRCPT1, R0 1.10566 1.22248 226 1489.35897 0.000

TIME slope, R1 0.08398 0.00705 226 261.20150 0.054

level-1, E 0.75948 0.57681

-----------------------------------------------------------------------------

Note: The chi-square statistics reported above are based on only 232 of 264

units that had sufficient data for computation. Fixed effects and variance

components are based on all the data.

Statistics for current covariance components model

--------------------------------------------------

Deviance = 2063.113251

Number of estimated parameters = 4

**Interaction Output**

Program: HLM 6 Hierarchical Linear and Nonlinear Modeling

Authors: Stephen Raudenbush, Tony Bryk, & Richard Congdon

Publisher: Scientific Software International, Inc. (c) 2000

techsupport@ssicentral.com

www.ssicentral.com

-------------------------------------------------------------------------------

Module: HLM2.EXE (6.08.29257.1)

Date: 30 June 2020, Tuesday

Time: 11:35:20

-------------------------------------------------------------------------------

SPECIFICATIONS FOR THIS HLM2 RUN

Problem Title: no title

The data source for this run = SC_Health.mdm

The command file for this run = C:\Users\heath\AppData\Local\Temp\whlmtemp.hlm

Output file name = C:\Users\heath\Dropbox\hlm2.txt

The maximum number of level-1 units = 792

The maximum number of level-2 units = 264

The maximum number of iterations = 100

Method of estimation: restricted maximum likelihood

Weighting Specification

-----------------------

Weight

Variable

Weighting? Name Normalized?

Level 1 no

Level 2 no

Precision no

The outcome variable is DHSYMP

The model specified for the fixed effects was:

----------------------------------------------------

Level-1 Level-2

Coefficients Predictors

---------------------- ---------------

INTRCPT1, P0 INTRCPT2, B00

ZAGE_6, B01

ZSEX_6, B02

ZSES_M6, B03

ZAVGSC, B04

ZBMI_O6_, B05

AGEX8SC, B06

* TIME slope, P1 INTRCPT2, B10

ZAGE_6, B11

ZSEX_6, B12

ZSES_M6, B13

ZAVGSC, B14

ZBMI_O6_, B15

AGEX8SC, B16

'*' - This level-1 predictor has been centered around its group mean.

The model specified for the covariance components was:

---------------------------------------------------------

Sigma squared (constant across level-2 units)

Tau dimensions

INTRCPT1

TIME slope

Summary of the model specified (in equation format)

---------------------------------------------------

Level-1 Model

Y = P0 + P1*(TIME) + E

Level-2 Model

P0 = B00 + B01*(ZAGE_6) + B02*(ZSEX_6) + B03*(ZSES_M6) + B04*(ZAVGSC)

+ B05*(ZBMI_O6_) + B06*(AGEX8SC) + R0

P1 = B10 + B11*(ZAGE_6) + B12*(ZSEX_6) + B13*(ZSES_M6) + B14*(ZAVGSC)

+ B15*(ZBMI_O6_) + B16*(AGEX8SC) + R1

Run-time deletion has reduced the number of level-1 records to 666

Iterations stopped due to small change in likelihood function

******* ITERATION 524 *******

Sigma_squared = 0.57563

Tau

INTRCPT1,P0 1.20864 0.01303

TIME,P1 0.01303 0.00709

Tau (as correlations)

INTRCPT1,P0 1.000 0.141

TIME,P1 0.141 1.000

----------------------------------------------------

Random level-1 coefficient Reliability estimate

----------------------------------------------------

INTRCPT1, P0 0.848

TIME, P1 0.074

----------------------------------------------------

Note: The reliability estimates reported above are based on only 232 of 264

units that had sufficient data for computation. Fixed effects and variance

components are based on all the data.

The value of the likelihood function at iteration 524 = -1.033461E+003

The outcome variable is DHSYMP

Final estimation of fixed effects:

----------------------------------------------------------------------------

Standard Approx.

Fixed Effect Coefficient Error T-ratio d.f. P-value

----------------------------------------------------------------------------

For INTRCPT1, P0

INTRCPT2, B00 1.225426 0.074410 16.469 257 0.000

ZAGE_6, B01 0.225087 0.076714 2.934 257 0.004

ZSEX_6, B02 0.213175 0.076304 2.794 257 0.006

ZSES_M6, B03 -0.110802 0.078627 -1.409 257 0.160

ZAVGSC, B04 -0.201770 0.076658 -2.632 257 0.009

ZBMI_O6_, B05 0.115734 0.075191 1.539 257 0.125

AGEX8SC, B06 -0.145764 0.077556 -1.879 257 0.061

For TIME slope, P1

INTRCPT2, B10 0.011312 0.020647 0.548 257 0.584

ZAGE_6, B11 0.029281 0.021377 1.370 257 0.172

ZSEX_6, B12 -0.002279 0.020959 -0.109 257 0.914

ZSES_M6, B13 0.007516 0.021415 0.351 257 0.726

ZAVGSC, B14 0.005738 0.021110 0.272 257 0.786

ZBMI_O6_, B15 0.023690 0.019826 1.195 257 0.234

AGEX8SC, B16 0.027408 0.021310 1.286 257 0.200

----------------------------------------------------------------------------

The outcome variable is DHSYMP

Final estimation of fixed effects

(with robust standard errors)

----------------------------------------------------------------------------

Standard Approx.

Fixed Effect Coefficient Error T-ratio d.f. P-value

----------------------------------------------------------------------------

For INTRCPT1, P0

INTRCPT2, B00 1.225426 0.074111 16.535 257 0.000

ZAGE_6, B01 0.225087 0.067629 3.328 257 0.001

ZSEX_6, B02 0.213175 0.069858 3.052 257 0.003

ZSES_M6, B03 -0.110802 0.074914 -1.479 257 0.140

ZAVGSC, B04 -0.201770 0.073100 -2.760 257 0.007

ZBMI_O6_, B05 0.115734 0.077773 1.488 257 0.138

AGEX8SC, B06 -0.145764 0.075372 -1.934 257 0.054

For TIME slope, P1

INTRCPT2, B10 0.011312 0.020359 0.556 257 0.579

ZAGE_6, B11 0.029281 0.018758 1.561 257 0.119

ZSEX_6, B12 -0.002279 0.020394 -0.112 257 0.912

ZSES_M6, B13 0.007516 0.023108 0.325 257 0.745

ZAVGSC, B14 0.005738 0.023464 0.245 257 0.807

ZBMI_O6_, B15 0.023690 0.017193 1.378 257 0.170

AGEX8SC, B16 0.027408 0.021349 1.284 257 0.201

----------------------------------------------------------------------------

Final estimation of variance components:

-----------------------------------------------------------------------------

Random Effect Standard Variance df Chi-square P-value

Deviation Component

-----------------------------------------------------------------------------

INTRCPT1, R0 1.09938 1.20864 225 1471.86026 0.000

TIME slope, R1 0.08423 0.00709 225 259.91794 0.055

level-1, E 0.75870 0.57563

-----------------------------------------------------------------------------

Note: The chi-square statistics reported above are based on only 232 of 264

units that had sufficient data for computation. Fixed effects and variance

components are based on all the data.

Statistics for current covariance components model

--------------------------------------------------

Deviance = 2066.922638

Number of estimated parameters = 4
